# Changes in Body Weight, Physical Activity, and Lifestyle During the Semi-lockdown Period After the Outbreak of COVID-19 in China: An Online Survey

**DOI:** 10.1017/dmp.2020.237

**Published:** 2020-07-14

**Authors:** Ming He, Yin Xian, Xiaodong Lv, Jinsong He, Yixing Ren

**Affiliations:** Department of General Surgery, and Institute of Hepato-Biliary-Pancreas and Intestinal Disease, Afﬁliated Hospital of North Sichuan Medical College, Nanchong, P.R. China

**Keywords:** body weight, coronavirus, lifestyle, lockdown, physical activity

## Abstract

**Objectives:**

The outbreak of novel coronavirus (COVID-19) provided an opportunity to undertake an online survey to study the relationships between body weight changes with changes in physical activity and lifestyle during an unusual event of forced isolation, or quarantine.

**Methods:**

We distributed an electronic questionnaire using the popular social application WeChat to adults from any province of China except Hubei Province, the epicenter of the outbreak. The questionnaire asked for demographic information, body weight, physical activity, and lifestyle factors before and during the quarantine.

**Results:**

Of 376 questionnaires returned, 339 were valid (90.2%). During the period of semi-lockdown, both females and males with BMI <24 gained weight, males with BMI ≥24 lost weight, and females with BMI ≥24 gained weight. The average steps per day and the average moderate or vigorous-intensity exercise declined significantly for both males and females during the semi-lockdown. Changes in body weight inversely correlated with changes in steps per day and moderate or vigorous-intensity exercise during the quarantine.

**Conclusions:**

Normal weight individuals, who are not normally troubled by overweight or obesity, had less awareness of weight gain than people with a BMI ≥ 24. Under the conditions of the semi-lockdown, they tended to gain weight.

Following the outbreak of coronavirus disease 2019 (COVID-19) caused by the novel coronavirus severe acute respiratory syndrome coronavirus-2 (SARS-CoV-2) in Wuhan in December 2019, the Chinese government announced a lockdown of Wuhan city on January 23, 2020, as part of a second stage of prevention and control measures to slow the spread of the epidemic.^1^ By January 29, control measures had been activated in all of China’s provinces, and a semi-lockdown was in place in several prefecture-level cities by February 8.^2^ Chinese New Year celebrations were canceled, collective activities, bus and railway service was suspended, and factories and restaurants were closed. Curfew and quarantine measures were implemented in many mainland cities. The flow of people was controlled by allowing only 1 person from each household to go out to buy necessities every 2-3 d. All of these measures to reduce transmission of the virus proved effective because by early March, the number of new cases was declining. The WHO-China Joint Mission on Coronavirus Disease 2019, conducted from February 16 to 24, 2020, reported that the country was beginning to return to normal.

In addition to the closures, the months of January and February are also the time of the Spring Festival in China, when people tend to consume high fat and calorie food more than usual, which might also contribute to changes in anthropometric measures. A retrospective cohort study showed that BMI of preschool children in 3 affected prefectures increased immediately after Great East Japan earthquake in 2011.^3^ Anticipating that the reduced physical activity resulting from the enforced quarantine would have an effect on anthropometric measures such as body weight, we undertook an online survey using the popular social media application WeChat during the month of March immediately after people began to return to normal.

## METHODS

This online survey was distributed as an electronic questionnaire through the social media platform WeChat from the Affiliated Hospital of North Sichuan Medical College, Sichuan Province, China, in March 2020. Participants were asked to complete the electronic questionnaire. Respondents were restricted to adults from any province of China except Hubei Province, the epicenter of the outbreak, which was under a much longer period of lockdown than other provinces.

The questionnaire designed by the authors was based on similar questionnaires reported in the literature.^4,5^ More modifications were made after receiving feedback from 31 early responses. The questionnaire consisted of 3 parts. The first part requested demographic and anthropometric information, including home domicile, gender, age, height, and weight changes (5 wk before and during the COVID-19 virus outbreak). From these data, we calculated body mass index (BMI), and the change in body weight from before until after the semi-lockdown. A high BMI for both males and females was a BMI ≥ 24. The second part requested information on participant lifestyle before the Epidemic Period of Coronavirus Disease 2019, such as the frequency of dinner parties of typically 8-10 people, amount of alcohol consumed during parties, the frequency of snacks, and lifestyle changes during period of semi-lockdown. The third part was about attitudes toward weight control and diet control (intention to control food intake, intention to remain physically active). We also collected data on exercise, including average number of walking steps and average of medium- or vigorous-intensity exercise time per day before (December 23, 2019, to January 26, 2020) and during (January 27, 2020, to March 1, 2020) semi-lockdown. The data for the third part were retrieved through Smartphone health software, such as Exercise Health or Keep. No financial and other material incentives were offered to the participants. We deployed the questionnaire on WeChat in early March and collected data until 20 March 2020.

### Data Analysis

Statistical data were analyzed by IBM SPSS version 25 (Armonk, NY: IBM Corp). The continuous variables are expressed as the median and interquartile range with the mean in graphs or as mean (standard deviation) in the tables. Shapiro-Wilk test and Kolmogorov-Smirnov test are used to test the normality of the continuity variables. The mean of independent samples was compared by independent sample t-test or the median by the Mann-Whitney test as necessary. The paired t-test and Wilcoxon signed-rank test were used to test differences between variables from before to after semi-lockdown. The chi-squared test was used to compare the differences of categorical variables. Spearman correlation was used to analyze the correlation between the other variables. *P* < 0.05 was considered statistically significant.

## RESULTS

### Participant Characteristics

Of 376 questionnaires returned, 339 were valid (90.2%). Thirty-seven questionnaires were not completed or the respondent was from Hubei province. Most of the responses were from Sichuan Province and from a few other provinces that were under semi-lockdown from January 27, 2020, to March 1, 2020. The 339 respondents included 158 males with a mean (SD) age of 36.4 (11.9) y and median age of 35 y and 181 females with a mean (SD) age of 37.6 (12.4) y and median age of 38 y.

### Change of Body Weight and Physical Activity

**FIGURE 1:**
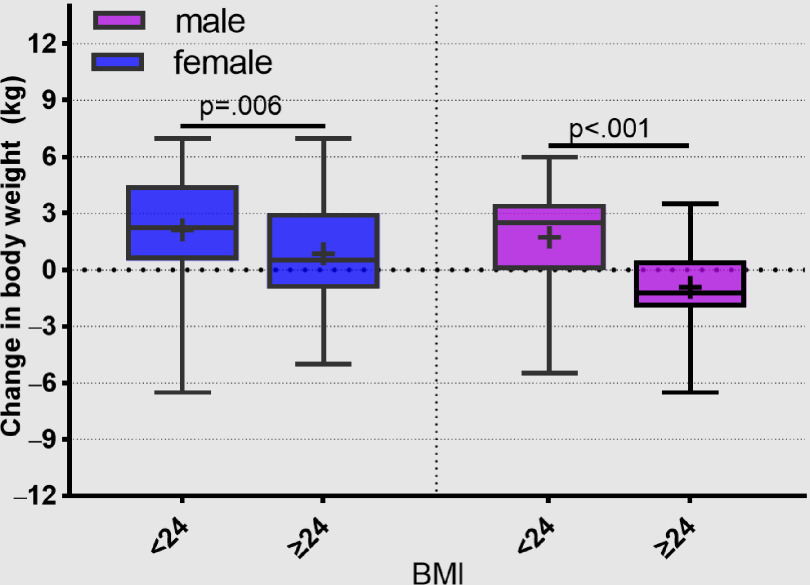
Change in Body Weight by BMI Category for Males and Females During the Period of Semi-lockdown (Median, Interquartile Range, Mean).

During the period of semi-lockdown, both females and males with BMI < 24 gained weight (before vs after 51.1 ± 4.1 vs 53.3 ± 5.9 kg, *P* < 0.001 and 65.6 ± 5.8 vs 67.3 ± 5.5 kg, *P* < 0.001 for female and male, respectively), and males with BMI ≥ 24 lost weight and females with BMI ≥ 24 gained weight (before vs after 62.9 ± 4.2 vs 63.8 ± 5.4 kg, *P* = 0.042 and 77.3 ± 7.5 vs 76.4 ± 6.9 kg, *P* = 0.003 for female and male, respectively) (Table 1). The change in body weight by BMI category during the period of semi-lockdown was significant for males and females (Figure 1).

**TABLE 1:** Body Weight Before and After the First 5 wk of the COVID-19 Outbreak by Gender and BMI Category

Gender	BMI Category	Body Weight Before (kg)	Body Weight After (kg)	*P*-Value
Male	BMI<24 (n = 110)	65.6 (5.8), 66.0	67.3 (5.5), 67	**<0.001**
	BMI≥24 (n = 48)	77.3 (7.5), 75.2	76.4 (6.9), 75	**0.003**
Female	BMI<24 (n = 128)	51.1 (4.1), 50.5	53.3 (5.9), 52.8	**<0.001**
	BMI≥24 (n = 53)	62.9 (4.2), 62	63.8 (5.4), 64.0	**0.042**

Values are mean (SD), median. Statistical comparison by paired t test. Bolding indicates statistical significance.

**FIGURE 2:**
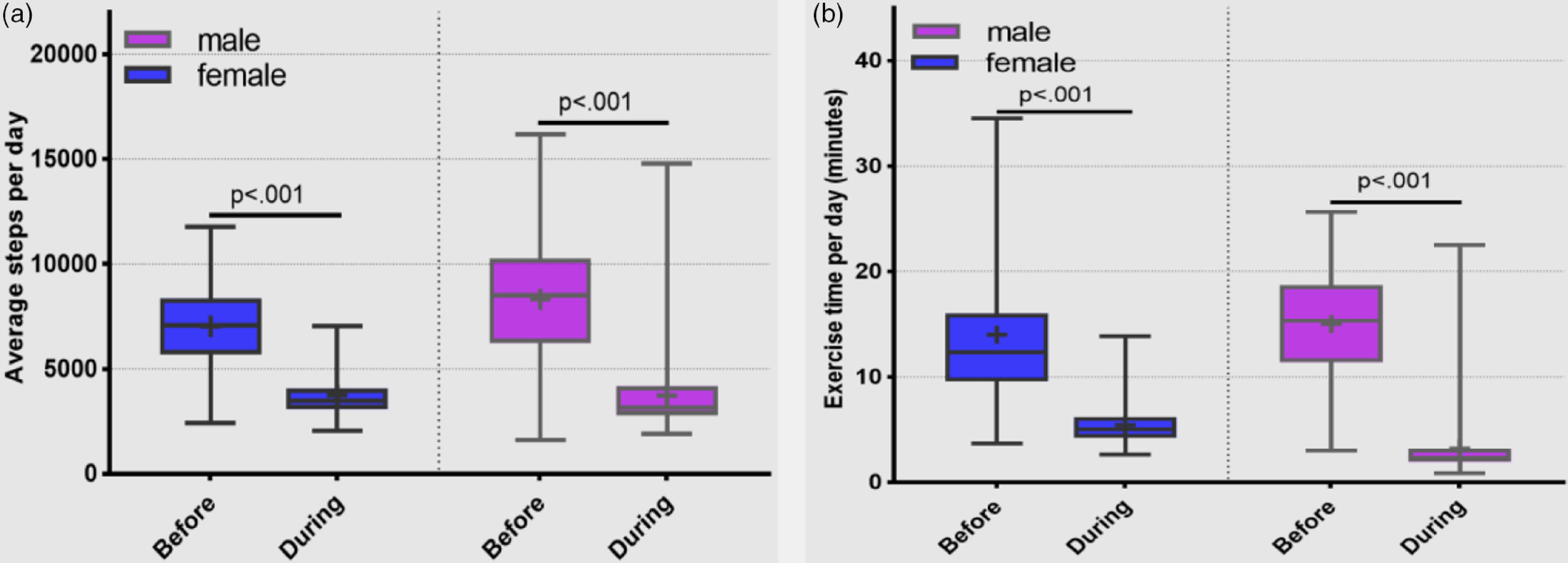
(a) Average steps per day before and during the period of semi-lockdown by gender (statistical comparison by paired t test). (b) Average moderate- or vigorous-intensity exercise (minutes per day) before and during the period of semi-lockdown by gender.

**FIGURE 3:**
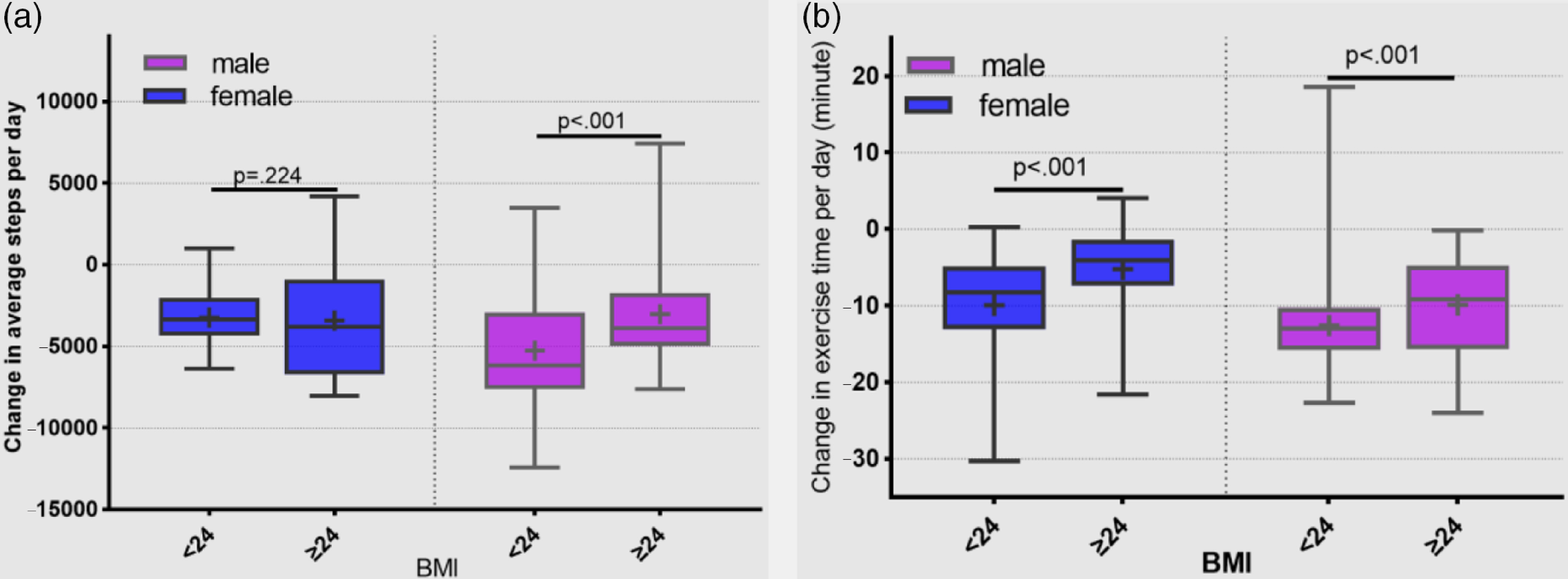
(a) Change in average steps per day by BMI category for males and females from before January 27 to during first 5 wk after January 27. (b) Change in moderate- or vigorous-intensity exercise (minutes per day) by BMI category for males and females from before January 27 to during first 5 wk after January 27 (median, interquartile range, mean).

The average steps per day and the average moderate- or vigorous-intensity exercise time declined significantly for both males and females during the period of semi-lockdown (average steps per day: before vs during 7038 ± 1923 vs 3741 ± 1042 step, *P* < 0.001 and 8321 ± 3000 vs 3728 ± 1726 step, *P* < 0.001 for female and male, respectively). The average moderate- or vigorous-intensity exercise time before vs during was 14.0 ± 6.3 vs 5.4 ± 2.0 min, *P* < 0.001 and 15.0 ± 5.1 vs 3.2 ± 3.2 min, *P* < 0.001 for female and male, respectively (Figure 2). The change in average steps per day by BMI category was significantly different for males but not for females. The change in moderate- or vigorous-intensity exercise was also significantly different by BMI category, with people of high BMI decreasing less than people of low BMI (Figure 3).

Statistical comparison by Wilcoxon signed rank test.

### Weight Control Awareness of Different BMI Categories

Before the COVID-19 epidemic, more people of high BMI intended to control food intake and increase physical activity than people with low BMI. Rate of intention to control food intake: BMI ≥ 24 vs BMI < 24. 67.9 vs 51.6%, *P* = 0.043 and 62.5 vs 43.6%, *P* = 0.029 for female and male, respectively. Rate of intention to increase physical activity: BMI ≥ 24 vs BMI < 24. 32.1 vs 18.0%, *P* = 0.037 and 27.1 vs 12.7%, *P* = 0.027 for female and male, respectively (Table 2).

**TABLE 2:** Intention to Change Lifestyle Factors by BMI Category for Males and Females Before the COVID-19 Epidemic

	Male (n = 158)			Female (n = 181)		
	**BMI <24 (n = 110)**	**BMI ≥24 (n = 48)**	***P*** **-Value**	**BMI<24 (n = 128)**	**BMI ≥24 (n = 53)**	***P*** **-Value**
Rate of intention to control food intake (%)	43.6	62.5	**.029**	51.6	67.9	**0.043**
Rate of intention to increase physical activity (%)	12.7	27.1	**.027**	18.0	32.1	**0.037**

Statistical comparisons by chi-squared test. Bolding indicates statistical significance.

### Influencing Factors on BMI

BMI before epidemic control of male residents was negatively correlated with the average steps per day (Rs = −0.336; *P* < 0.001), the average moderate- or vigorous-intensity exercise minutes (Rs = −0.329; *P* < 0.001), and positively correlated with the frequency of dinner parties (Rs = 0.184, *P* = 0.021), the alcohol consumption during dinner parties (Rs = 0.161; *P* = 0.044). The BMI before epidemic control of female was negatively correlated with the average moderate- or vigorous-intensity exercise minutes and positively correlated with the frequency of snacks (Rs = −0.215; *P* = 0.004 and Rs = 0.186, *P* = 0.012, respectively) (Table 3).

**TABLE 3:** Correlation Analysis BMI Before Epidemic Control With Physical Activity and Lifestyle (Spearman Correlation)

Parameters	BMI Before Epidemic Control			
	Male (n = 158)		Female (n = 181)	
	R_S_	*P*-Value	R_S_	*P*-Value
Average steps per day	−0.336	**<0.001**	−0.006	0.938
Average moderate- or vigorous-intensity exercise per day	−0.329	**<0.001**	−0.215	0.**004**
Frequency of dinner parties	0.184	0.**021**	−0.121	0.106
Drinking volume while Dining	0.161	0.**044**	−0.003	0.967
Frequency of snacks	0.055	0.495	0.186	0.**012**

Bolding indicates statistical significance.

### Influencing Factors on the Changes in Body Weight

**FIGURE 4:**
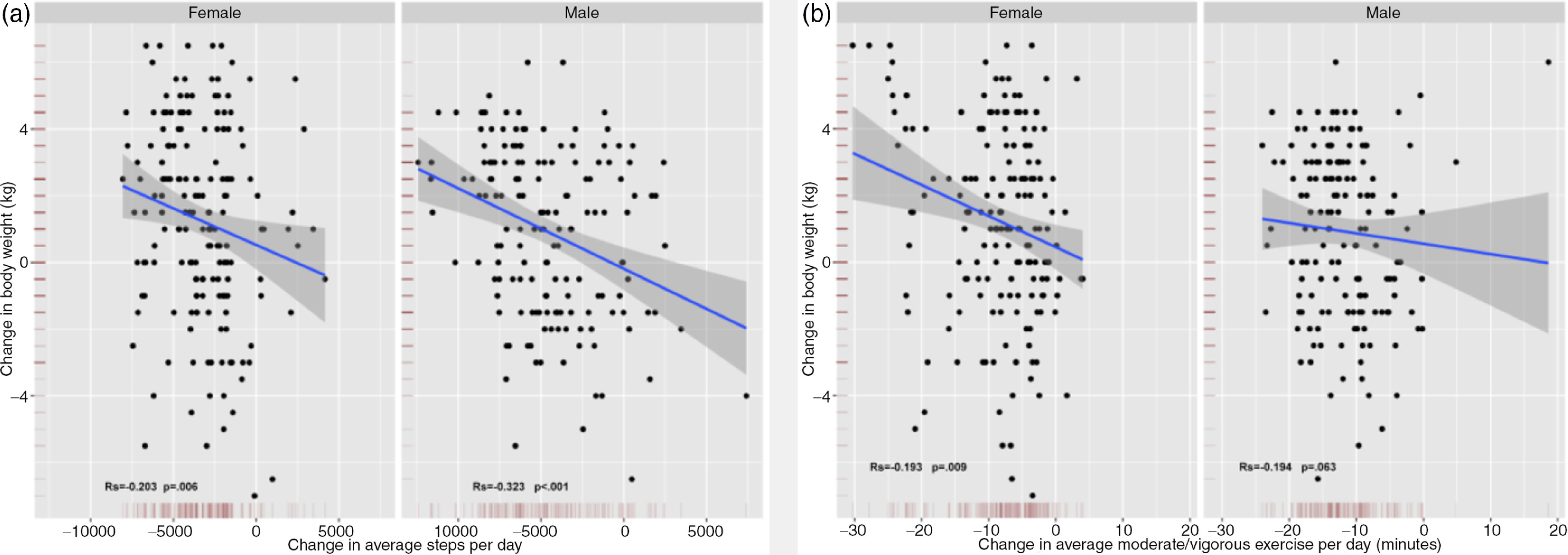
(a) Change in average steps per day by gender by change in body weight. (b) Change in average moderate- or vigorous-intensity exercise per day by gender by change in body weight.

The changes in body weight inversely correlated with changes in steps per day and moderate- or vigorous-intensity exercise in males and females and were statistically significant, except for males for moderate- or vigorous-intensity exercise time (Figure 4). Changes in body weight of males were inversely correlated with the change level of alcohol consumption during the semi-lockdown for COVID-19 (Rs = −0.255; *P* = 0.002).

Spearman correlation analysis.

## DISCUSSION

Obesity is a pathological state related to both genetic and acquired factors, particularly exercise and diet.^6^ Important reasons for the prevalence of obesity are consumption of energy-rich foods and a lack of exercise.^7^ Because of the rarity of events such as the recent outbreak of novel coronavirus, the effects of a period of isolation at home have not been well studied. Because the lifestyles of Chinese residents were completely altered by this rare event, we recognized the opportunity to conduct a survey to determine the effects of the quarantine on weight changes. Surprisingly, we found that, after the first 5 wk epidemic period of COVID-19, both females and males with BMI < 24 gained weight, male residents with BMI ≥ 24 lost weight, and females with a BMI ≥ 24 gained weight. BMI before the epidemic control was positively correlated with the frequency of dinner parties of several people and with the consumption of alcohol while dining, particularly in males. In China, the food consumed during dinner parties is often high-fat and high calorie. During the period of semi-lockdown, government rules disallowed people from gathering for dinner parties in home or at restaurants. So, the decrease in energy and alcohol intake during the semi-lockdown, when people were unable to eat at normal levels of consumption, leads to significant weight loss.

This finding is consistent with reports that intermittent energy restriction or persistent energy restriction can help reduce BMI,^8^ and that the relationship between alcohol consumption and obesity is linear with nondrinkers having a low risk of obesity.^9^ According to the Korea National Health and Nutrition Examination Survey, a cross-sectional and nationally representative survey conducted from 1998 to 2012 in South Korea, an increase in the risk of obesity was accompanied by an overconsumption of energy in men (but not women), and by low physical activity and high-risk drinking by both sexes.^10^ Based on this survey, we consider that, for weight changes, the effect of weight loss due to reduced calorie intake was more powerful than weight gain due to reduced physical activity.

In our study, we evaluated respondents’ awareness of weight control by asking whether they usually intended to control food intake and increase physical activity. Men with BMI ≥ 24 had a greater awareness of weight control than men with BMI < 24, but were often unable to effectively reduce social intercourse and drinking due to work or other reasons, which resulted in overweight or obesity. The control period following the COVID-19 outbreak was actually conducive to weight control because of the interference with social intercourse.

The reasons for weight gain in males and females with BMI < 24 are explained by the correlation analysis that indicated that before the epidemic, the normal weight population engaged in more physical activity, attended dinner parties less frequently, and consumed less alcohol compared with the overweight or obese population, so the change in physical activity was the most obvious during the period of semi-lockdown, which resulted in weight gain. This is consistent with the findings of the China Kadoorie Biobank study and the European Kardiovize Brno 2030 Study in which greater physical activity was related to a lower BMI.^11,12^ In our study, people with a BMI < 24 were not normally troubled by overweight or obesity, but also had less awareness of weight gain than people with a BMI ≥ 24. Therefore, under the conditions of the semi-lockdown, they tended to gain weight more easily.

We also found that females with a BMI ≥ 24 gained less weight than females with a BMI < 24. The change in the average daily physical activity of overweight or obese residents was less than that of normal weight people during the period of control. Because the rise in BMI before epidemic control was negatively correlated with the average daily moderate- or vigorous-intensity exercise minutes, overweight or obese people exercised less in normal times, leading to females with BMI ≥ 24 gaining less weight and males with BMI ≥ 24 losing weight after home isolation.

The limitations of our study are the relatively small sample size and short 5-wk period of the study. Some provinces in China cancelled the first level response to the public health emergency and some industries have resumed production, so the flow of personnel began to increase, which may have caused weight changes to eb affected by other factors. There are certain limitations using the bivariate analyses. In the further research, we need to involve more sample size and independent variables for multivariate analysis.

## CONCLUSIONS

In conclusion, the virus that causes COVID-19 is still prevalent all over the world. Some countries have gradually adopted forced isolation measures such as lockdown and reducing the flow of people. Such measures force changes in lifestyle, which may lead to changes in body weight. Normal weight individuals may not normally be troubled by overweight or obesity and may have less awareness of weight gain than people with a BMI ≥ 24. Therefore, normal weight as well as overweight and obese people should be aware of the need for weight control when physical activity is necessarily reduced by epidemic prevention and control measures.
